# The efficacy and safety of triptorelin-therapy following conservative surgery for deep infiltrating endometriosis: A multicenter, prospective, non-interventional study in China

**DOI:** 10.1097/MD.0000000000028766

**Published:** 2022-02-04

**Authors:** Libo Zhu, Zheng Guan, Yan Huang, Keqin Hua, Liguo Ma, Jian Zhang, Dazhen Yang, Valerie Perrot, Hongbo Li, Xinmei Zhang

**Affiliations:** a Gynaecology Department, Women's Hospital, School of Medicine, Zhejiang University, Hangzhou, China; b Gynaecology Department, Chinese PLA General Hospital, Beijing, China; c Gynaecology Department, The Second Affiliated Hospital of Army Medical University, Chongqing, China; d Gynaecology Department, Obstetrics & Gynecology Hospital of Fudan University, Shanghai, China; e Gynaecology Department, Shenzhen People's Hospital, Guangdong Province, China; f Gynaecology Department, International Peace Maternity and Child Health Hospital of the China Welfare Institute, Shanghai, China; g Gynaecology Department, Nanjing Maternity and Child Health Care Hospital, Nanjing, Jiangsu Province, China; h Clinical Statistics Department, Ipsen, Boulogne-Billancourt, France; i Medical Affairs Department, Ipsen (Beijing) Pharmaceutical Science and Technology Development Co., Ltd., Beijing, China.

**Keywords:** deep infiltrating endometriosis, endometriosis symptoms, improvement, recurrence, triptorelin

## Abstract

Triptorelin is one of the most commonly used gonadotropin-releasing hormone agonists and has been used in the treatment of deep infiltrating endometriosis (DIE). This study aimed to evaluate the efficacy and safety of up to 24 weeks of triptorelin treatment after conservative surgery for DIE.

This prospective, non-interventional study was performed in 18 tertiary hospitals in China. Premenopausal women aged ≥18 years treated with triptorelin 3.75 mg once every 28 days for up to 24 weeks after conservative surgery for DIE were included. Endometriosis symptoms were assessed, using a visual analogue scale (0–10 cm) or numerical range (0–10), at baseline (pre-surgery) and routine visits 3, 6, 9, 12, 18, and 24 months after surgery. Changes in symptom intensity over time were primary outcome measures.

A total of 384 women (mean [standard deviation] age, 33.4 [6.2] years) were analyzed. Scores for all symptoms (pelvic pain, dysmenorrhea, ovulation pain, dyspareunia, menorrhagia, metrorrhagia, and gastrointestinal and urinary symptoms) assessed decreased from baseline over 24 months. Cumulative improvement rates in pelvic pain, dysmenorrhoa, ovulation pain, and dyspareunia were 74.4%, 83.6%, 55.1%, and 66.9%, respectively. The 24-month cumulative recurrence rate (≥1 symptom) was 22.2%. The risk of symptom recurrence was higher in patients with ≥2 versus 1 lesion (odds ratio [OR] 2.539; 95% CI: 1.458–4.423; *P* = .001) and patients with moderate (OR 5.733; 95% CI: 1.623–20.248; *P* = .007) or severe (OR 8.259; 95% CI: 2.449–27.851; *P* = .001) pain versus none/mild pain. Triptorelin was well tolerated without serious adverse events.

Triptorelin after conservative surgery for DIE improved symptoms over 24 months of follow up. The recurrence rate of symptoms was low and triptorelin was generally well tolerated.

Trial registration number: ClinicalTrials.gov, NCT01942369.

**Key Points**

- The first study focuses on the symptom control of post-operative triptorelin therapy in patients with deep infiltrating endometriosis conducted in multi-centers in China and suggests that triptorelin following conservative surgery was effective and well tolerated.

## 1 Introduction

Endometriosis is defined by the presence of endometrial-like tissue outside the uterus, most commonly on the peritoneal and visceral surfaces of the pelvis, which triggers a chronic inflammatory reaction.^[1]^ Women during the reproductive period are predominantly affected, with the main symptoms being pain (i.e., ovulation pain, dysmenorrhea, dyspareunia, and chronic pelvic/abdominal pain) and infertility.^[1–3]^ The overall prevalence of endometriosis in women of reproductive age is estimated at 2% to 10%.^[4,5]^ However, the prevalence of endometriosis is 71% to 87% among women suffering from chronic pelvic pain and 50% among infertile women.^[6,7]^

Endometriosis can be divided into 3 types based on severity: superficial peritoneal endometriosis (least severe), ovarian endometriosis, and deep infiltrating endometriosis (DIE, most severe; also known as deep endometriosis, or DE).^[8,9]^ DIE is the most aggressive of the 3 clinical forms, characterized by deep infiltration and the most severe pain symptoms. Furthermore, it is the most difficult to manage and is associated with high direct and indirect treatment costs.^[10]^

Surgery is the preferred treatment in cases of DIE and the effectiveness of conservative surgery in patients with DIE has been demonstrated.^[11,12]^ However, for one thing, surgery might not completely eliminate the lesion, residual lesions, and new lesions may continue to grow or develop. For another, surgery is associated with the development of pelvic cavity adhesions. Additionally, the reported 3-year symptom recurrence rate following surgery was 38% to 51%.^[13]^

A variety of medical therapies are now available for symptomatic endometriosis.^[14]^ Gonadotropin-releasing hormone agonists (GnRH-a) have been recommended as second-line therapies for improving symptoms.^[10]^ Triptorelin is one of the most commonly used GnRH-a and has been demonstrated to improve symptoms and fertility outcomes of patients with DIE.^[15,16]^

Although multiple studies have shown that GnRH-a treatment following surgery can improve postoperative pain and may facilitate a reduction in the rate of symptom recurrence,^[17–19]^ the effectiveness and optimal duration of GnRH-a treatment after surgery in DIE are still debated in China. This study aimed to evaluate whether up to 24 weeks of treatment with triptorelin following conservative surgery can help manage the DIE symptoms, reduce symptom recurrence rate, and improve fertility status over a 24-month follow-up period in clinical practice in China.

## 2 Materials and methods

This multicenter, prospective, non-interventional, observational study was conducted in 18 tertiary hospitals in China between September 8, 2013 and July 13, 2018. The study was approved by the Institutional Review Board at Women's Hospital, School of Medicine, Zhejiang University and by ethical committees of all study centers and was registered at www.clinicaltrials.gov (NCT01942369).

### 2.1 Patients

Patients were screened using electronic diagnostic records and were eligible according to the following inclusion criteria: patients who were premenopausal women aged ≥18 years with a clinical diagnosis of DIE; had received conservative surgery within 1 month of the study start date; had been prescribed triptorelin; and were mentally and physically able to describe their symptoms and answer questions. Conservative surgery for the treatment of DIE refers to the removal or destruction endometriosis lesions or nodules, separation of adhesions, removal of ovarian chocolate cyst, and preservation of uterus and ovary, so as to preserve the reproductive function. Exclusion criteria: pregnant or lactating women; premenopausal women who may reach menopause within 3 years of surgery; a history of allergic reaction to triptorelin or one of the excipients; a history of treatment with other drugs within 3 months and GnRH-a therapy within 6 months prior to the study; and women who were potentially non-compliant or unsuitable for the study for other reasons, as judged by the investigator.

All included patients provided written, informed consent.

### 2.2 Treatment and follow up

Patients received triptorelin acetate 3.75 mg as an intramuscular injection (Diphereline, Ipsen) once in the cycle of 28 days for up to 24 weeks (≤6 injections). The prescription and number of required treatment cycles were determined by physicians based on routine clinical practice, without external intervention. Moreover, other treatments and management of adverse events were in accordance with routine clinical practice.

Patients were assessed at baseline (pre-surgery) and then at routine post-operative hospital visits every 3 months in the first year of follow up and every 6 months in the second year of follow up. At each visit, data and information of endometriosis symptoms and pregnancy status, as well as imaging and laboratory examinations were collected.

### 2.3 Efficacy outcomes

In the Core Outcomes in Women's and Newborn Health database, a core outcome set for endometriosis is currently in development. The primary outcome was changes in intensity/severity of endometriosis symptoms from baseline (pre-surgery) to 24-month follow up. Symptoms of pelvic pain, dysmenorrhea, pain at the time of ovulation, and dyspareunia were assessed using a visual analogue scale (VAS) with a range from 0 to 10 cm, and symptoms of amenorrhea, menorrhagia, metrorrhagia, and gastrointestinal and urinary discomfort were assessed using a numerical scale ranging from 0 to 10. A score of 0 indicated that the patient was free of pain or symptoms, while a score of 10 represented pain or symptoms that were severe and intolerable. Symptoms with a VAS or numerical score >0 and ≤3, >3 and <7, and ≥7 were considered as mild, moderate, and severe, respectively. An improvement in symptoms was defined as a reduction of at least 3 cm or 3 units from presurgery levels. The proportions of patients with symptoms overall and with symptoms of different intensity/severity levels were also assessed.

Secondary efficacy outcomes were the rates of symptom recurrence and pregnancy. Symptom recurrence was defined as an increase of >3 cm or 3 units on the VAS or numerical score compared with the lowest previous score.

### 2.4 Exploratory analyses

Exploratory analyses of predictive factors for both symptom recurrence and the duration of triptorelin treatment were also conducted. For symptom recurrence, factors evaluated included age, body mass index, duration of medical attention, reproductive status, comorbidities, history of surgery, surgical procedures, the site and number of DIE lesions, pain prior to surgery, pain intensity, number of triptorelin doses, and concomitant medications after GnRHa. Predictive factors associated with treatment duration included age at surgery, DIE lesion, previous surgical diagnosis of endometriosis, previous hormonal treatment for endometriosis, history of pregnancy, intensity of dyspareunia, dysmenorrhea, menorrhagia, gastrointestinal, urinary, and pain symptoms prior to surgery and previously infertility.

### 2.5 Safety outcomes

All spontaneously reported adverse events were collected up to 1 month after the last triptorelin injection.

### 2.6 Statistical analysis

Baseline characteristics were assessed with descriptive statistical analysis; the mean ± standard deviation (SD) or median (range) are presented for continuous variables and frequency distribution (n, %) presented for categorical variables. Evolution of symptoms intensity scores and proportion (%) of patients are presented. Logistic regression was used to estimate the cumulative improvement and recurrence rates.

Univariate and multivariate binary logistic regression models controlling for baseline characteristics and numbers of triptorelin injections were used to assess risk factors for symptom recurrence. Univariate and multivariate Cox proportional regression models controlling for baseline demographics and clinical characteristics were used to identify factors associated with triptorelin treatment duration. The factors significant at 20% (*P* < .20) in the univariate analysis were entered in the multivariate model. All tests were 2 sided and a level of *P* < .05 was considered as statistically significant. All statistical analysis was performed in SAS version 9.21 (SAS Institute, Cary, NC).

## 3 Results

### 3.1 Patients

**Figure 1:**
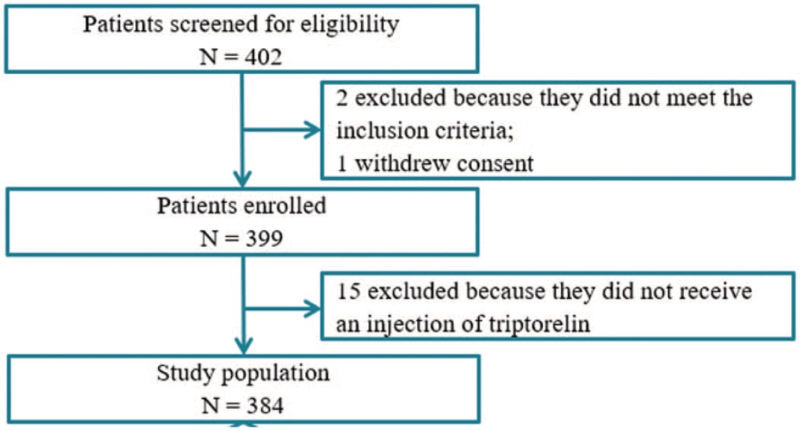
Flow diagram of study population.

In total, 402 patients were screened for the study. One of them was excluded because of informed consent withdrawn and 2 were excluded because they did not meet the inclusion criteria. Consequently, 399 were enrolled with written informed consent and 384 (96.2%) patients with triptorelin-therapy following conservative surgery for DIE were included in the final analysis (Fig. 1). Fifteen patients were excluded from the final analysis because of missing data on triptorelin administration.

The baseline characteristics of patients are shown in Table 1. The mean age of patients was 33.4 ± 6.2 years; 80 (20.8%) patients had received a previous surgical diagnosis of endometriosis, 4 (5.0%) of them had received previous surgery for DIE. Overall, 63 (16.4%) patients had received a previous medical treatment for endometriosis.

**Table 1:** Baseline demographics and clinical characteristics of study population.

Characteristics	Total (N = 384)
Age, y, mean ± SD	33.4 ± 6.2
BMI, kg/m^2^, mean ± SD	21.0 ± 2.8
Age when first medical attention sought, y, (n = 327), mean ± SD	31.4 ± 6.3
Age at first endometriosis surgical diagnosis, y (n = 80), mean ± SD	29.8 ± 5.3
Location of DIE lesions, n (%)	
Intestine	213 (55.5)
Vagina	37 (9.6)
Ureter	10 (2.6)
Bladder	8 (2.1)
Left uterosacral ligament	42 (10.9)
Right uterosacral ligament	31 (8.1)
Bilateral uterosacral ligament	194 (50.5)
Treatment history	
Previously surgically diagnosed with endometriosis, n (%)	80 (20.8)
Previous surgery for endometriosis (other than DIE), n (%)	43 (53.8)
Previous surgery for DIE, n (%)	4 (5.0)
Previous medical treatment for endometriosis, n (%)	63 (16.4)
Oral contraceptive pills treatment duration, mo (n = 10), mean ± SD	15.2 ± 24.4
GnRH-a treatment duration, mo (n = 18), mean ± SD	3.9 ± 1.9
Progesterone treatment duration, mo (n = 5), mean ± SD	27.6 ± 25.9
Traditional Chinese medication treatment duration, mo (n = 38) (mean ± SD)	10.6 ± 15.2
Reproductive history, n (%)	
Previous pregnancy history	249 (64.8)
Previous miscarriage history^∗^	84 (33.7)
Previous voluntary abortion history^∗^	81 (32.5)
Previous therapeutic abortion history^∗^	35 (14.1)
Previous ectopic abortions^∗^	8 (3.2)

BMI = body mass index, DIE = deep infiltrating endometriosis, GnRH-a = gonadotropin-releasing hormone agonist.

∗ The proportions were calculated based on pregnant women.

During surgery, 149 (38.8%) patients displayed peritoneal superficial endometriosis and 357 (93.0%) had endometrioma. DIE lesions were located on the intestine, bilateral uterosacral ligament, vagina, ureter, and bladder in 55.5%, 50.5%, 9.6%, 2.6%, and 2.1% of patients, respectively. The majority of patients (64.8%) had a history of pregnancy. The proportion of history of miscarriage, voluntary abortion, therapeutic abortion, and ectopic abortions was 33.7%, 32.5%, 14.1%, and 3.2%, respectively.

A total of 35 (9.1%) patients received 1 to 2 injections of triptorelin, 191 (49.7%) received 3 to 5 injections, and 158 (41.2%) received 6 injections. The median duration of drug exposure was 13.0 weeks (range: 0–29 weeks).

### 3.2 Symptoms

**Figure 2:**
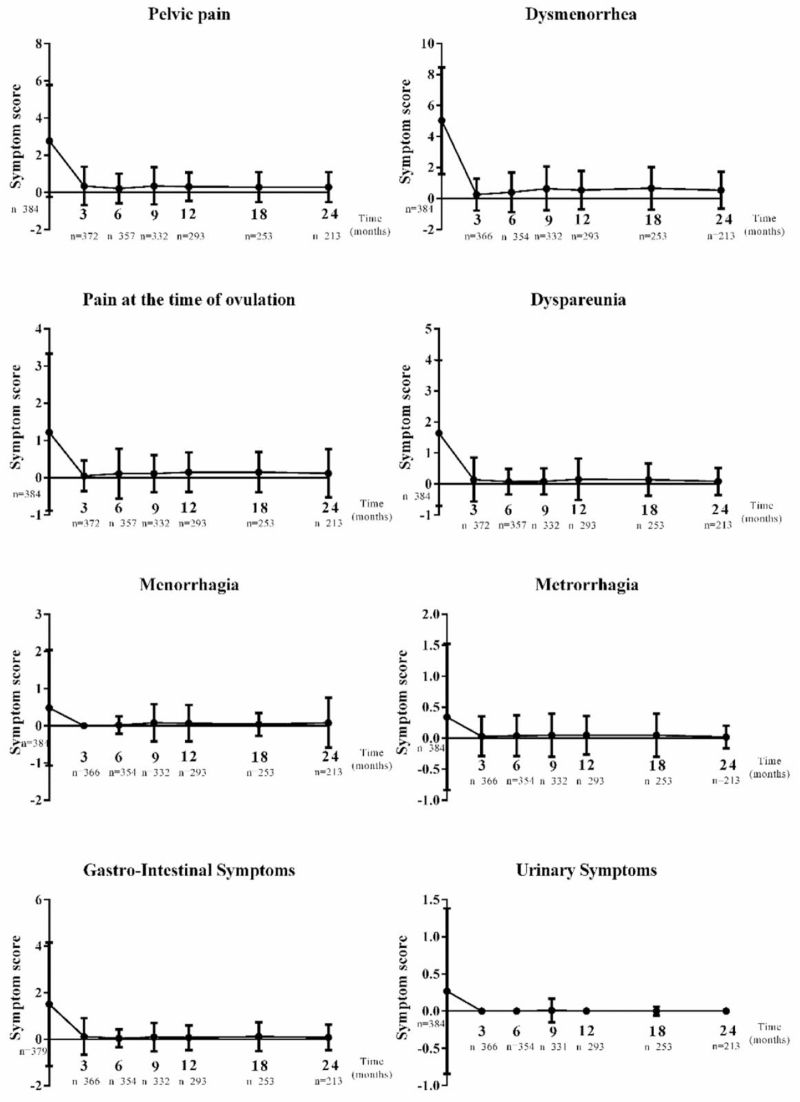
Changes in the intensity of endometriosis symptoms over time. A visual analogue scale (VAS) or a numerical scale was used to evaluate pain or other symptoms, respectively. A score of 0 indicated that the patient was free of pain or other symptoms while 10 indicated the pain or symptoms were severe and intolerable. Mean scores and standard deviations of symptom intensities against time are presented.

Changes in the intensity of symptoms over time from baseline (presurgery) to 24 months after surgery are summarized in Fig. 2. The scores for all symptoms decreased during follow up. At baseline and 24 months, respectively, symptom scores (mean [SD]) were: pelvic pain, 2.8 (3.0) and 0.3 (0.8); dysmenorrhea, 5.0 (3.4) and 0.5 (1.2); ovulation pain, 1.2 (2.1) and 0.1 (0.7); dyspareunia, 1.6 (2.4) and 0.1(0.4); gastrointestinal symptoms, 1.5 (2.7) and 0.1 (0.6).

**Figure 3:**
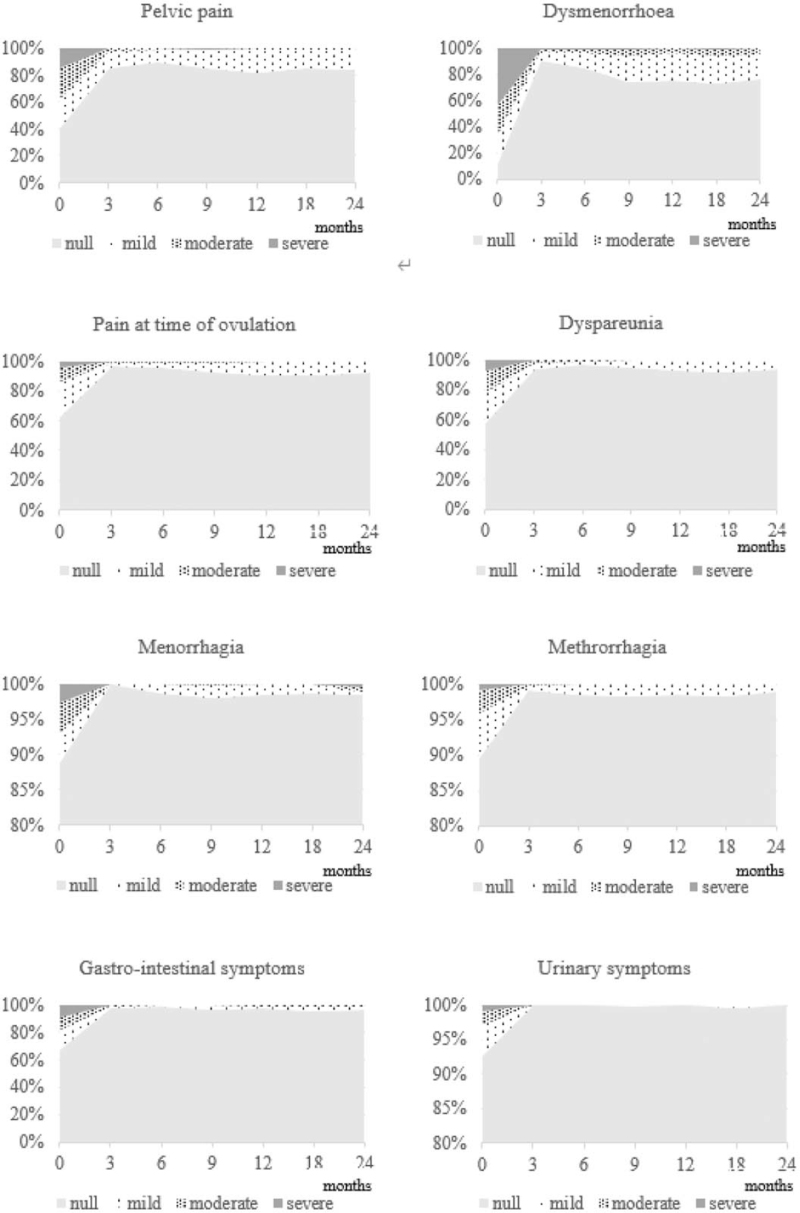
Changes in the proportions of patients with different intensities of endometriosis symptoms over time. Cumulative proportion of patients with different symptom intensities are shown against time. A VAS or numerical score ≥7 was considered as severe, a score of >3 and <7 as moderate, and a score of >0 and ≤3 as mild.

At baseline, the proportions of women reporting pelvic pain, dysmenorrhea, ovulation pain, dyspareunia, or gastrointestinal symptoms were 58.1% (223/384), 84.1% (323/384), 35.9% (138/384), 42.4% (163/384), and 31.4% (119/379), respectively; at 24 months, these proportions were reduced to 16.0% (34/213), 23.0% (49/213), 6.6% (14/213), 5.6% (12/213), and 2.8% (6/213), respectively (Fig. 3). From baseline to month 24, there were considerable reductions in the proportions of patients with severe pelvic pain (15.1 to 0.5%), dysmenorrhea (41.4 to 0.5%), ovulation pain (4.4 to 0.5%), dyspareunia (6.0 to 0%), and gastrointestinal symptoms (9.2 to 0%). Similarly, the proportions of patients with moderate symptoms were reduced from baseline over the 24 months for pelvic pain (21.9 to 0.5%), dysmenorrhea (21.4 to 3.8%), ovulation pain (8.9 to 0%), dyspareunia (14.8 to 0.5%), and gastrointestinal symptoms (8.4 to 0.9%).

At baseline, few reported menorrhagia (10.7%), metrorrhagia (9.6%), and urinary symptoms (7.6%) and the proportions decreased further from baseline to month 24 (Fig. 3).

Among patients with a symptom intensity score >0 at baseline, the cumulative improvement rates at 12 months were 72.6% for pelvic pain, 83.3% for dysmenorrhea, 55.1% for ovulatory pain, 66.9% for dyspareunia, 78.0% for menorrhagia, 59.5% for metrorrhagia, 75.6% for gastrointestinal symptoms, and 58.6% for urinary symptoms. Almost all improvements occurred in the first 12 months. Similar cumulative improvement rates were observed for all symptoms at 24 months (Table 2).

**Table 2:** Efficacy outcomes in patients treated with triptorelin for up to 24 weeks following conservative surgery for DIE.

	12 months		24 months	
Outcomes	N	n (%)	N	n (%)
Cumulative improvement rate				
Pelvic pain	223	162 (72.6)	223	166 (74.4)
Dysmenorrhea	323	269 (83.3)	323	270 (83.6)
Pain at time of ovulation	138	76 (55.1)	138	76 (55.1)
Dyspareunia	163	109 (66.9)	163	109 (66.9)
Menorrhagia	41	32 (78.0)	41	32 (78.0)
Metrorrhagia	37	22 (59.5)	37	22 (59.5)
Gastrointestinal symptoms	119	90 (75.6)	119	90 (75.6)
Urinary symptoms	29	17 (58.6)	29	17 (58.6)
Cumulative recurrence rate	320	41 (12.8)	320	71 (22.2)
Pelvic pain	320	10 (3.1)	320	14 (4.4)
Dysmenorrhea	320	32 (10.0)	320	54 (16.9)
Pain at time of ovulation	320	4 (1.3)	320	6 (1.9)
Dyspareunia	320	2 (0.6)	320	4 (1.3)
Menorrhagia	320	3 (0.9)	320	5 (1.6)
Metrorrhagia	320	0 (0)	320	0 (0)
Gastrointestinal symptoms	320	4 (1.3)	320	10 (3.1)
Urinary symptoms	320	0 (0.0)	320	0 (0.0)
Pregnancy rate, study population	384	42 (10.9)	384	66 (17.2)
Pregnancy rate, previously infertile patients	59	11 (18.6)	59	16 (27.1)
Spontaneous pregnancy	11	8 (72.7)	16	10 (62.5)
Assistance fertility	11	3 (27.3)	16	6 (37.5)

### 3.3 Symptom recurrence and pregnancy

The 24-month cumulative symptom recurrence rate, defined as ≥1 symptom of recurrence, was 22.2% (Table 2). The 12-month and 24-month cumulative recurrence rates, respectively, were 3.1% and 4.4% for pelvic pain, 10.0% and 16.9% for dysmenorrhea, 1.3% and 1.9% for ovulatory pain, 0.6% and 1.3% for dyspareunia, 0.9% and 1.6% for menorrhagia, 0% and 0% for metrorrhagia, 1.3% and 3.1% for gastrointestinal symptoms, and 0% and 0% for urinary symptoms.

In total, the 12-month and 24-month cumulative pregnancy rates were 10.9% and 17.2%, respectively. Among 59 patients diagnosed with infertility previously, the 12-month and 24-month cumulative pregnancy rates were 18.6% and 27.1%, respectively (Table 2).

### 3.4 Predictive factors for symptom recurrence

The univariate logistic regression analysis identified DIE lesion in the intestine or in uterosacral ligament, presurgery pain symptoms with or without gastrointestinal symptoms and with or without infertility, number of DIE lesion locations, intensity of pain, and number of doses of triptorelin (all *P* < .20) as the risk factors for symptom recurrence (Table 3). In the multivariate logistic regression model, the risk of symptom recurrence was significantly higher in patients with 2 or more lesions compared with those with 1 lesion (OR 2.539; 95% CI: 1.458–4.423; *P* = .001) and in patients with moderate pain (OR 5.733; 95% CI: 1.623–20.248; *P* = .007) or severe pain (OR 8.259; 95% CI: 2.449–27.851; *P* = .001) compared with those with none or mild pain.

**Table 3:** Predictive factors for symptom recurrence (logistic regression analysis).

	Univariate analysis		Multivariate analysis	
Factors	OR (95% CI)	*P* value	OR (95% CI)	*P* value
DIE lesion in intestine				
Yes vs no	2.072 (1.182–3.631)	.011		
DIE lesion in uterosacral ligament				
Yes vs no	1.627 (0.878–3.016)	.122		
Presurgery specific symptoms				
Pain with vs without GI symptoms	1.521 (0.824–2.809)	.180		
Pain with vs without infertility	0.174 (0.023–1.345)	.094		
Number of DIE lesion locations				
≥ 2 vs 1	2.621 (1.529–4.492)	<.001	2.539 (1.458–4.423)	.001
Intensity of pain (vs null/mild)				
Moderate	6.328 (1.809–22.140)	.004	5.7 (1.623–20.248)	.007
Severe	8.547 (2.554–28.601)	<.001	8.3 (2.449–27.851)	.001
Triptorelin treatment				
4–6 vs 1–3 doses	1.829 (1.029–3.251)	.040		

CI = confidence interval, DIE = deep infiltrating endometriosis, GI = gastrointestinal, OR = odds ratio.

### 3.5 Predictive factors for duration of triptorelin treatment

Age, associated DIE lesion, history of hormonal treatment for endometriosis, presurgery intensity of dysmenorrhea, ovulation pain, and gastrointestinal symptoms and infertility (all *P* < .20) were identified by the univariate Cox regression as predictive factors for triptorelin treatment duration (Table 4). In a multivariate Cox regression analysis, the duration of triptorelin treatment was longer in patients older versus younger at the time of surgery (hazard ratio [HR] 0.983; 95% CI: 0.968–1.000; *P* = .044), who previously received hormonal treatment for endometriosis compared with those who did not (HR 0.724; 95% CI:0.543–0.950; *P* = .024), and shorter in patients who were infertile versus fertile (HR 1.401; 95% CI:1.046–1.844; *P* = .019).

**Table 4:** Predictive factors for duration of triptorelin treatment (Cox regression analysis).

	Univariate analysis		Multivariate analysis	
Factors	HR (95% CI)	*P* value	HR (95% CI)	*P* value
Age at surgery, y	0.985 (0.970–1.001)	.064	0.983 (0.968–1.000)	.044^∗^
Associated DIE lesion				
Yes vs no	0.819 (0.650–1.033)	.092		
Previously surgically diagnosed with endometriosis				
Yes vs no	0.837 (0.653–1.072)	.158		
Previously treatment with a hormonal for endometriosis				
Yes vs no	0.748 (0.569–0.981)	.036^∗^	0.724 (0.543–0.950)	.024^∗^
Intensity of dysmenorrhea prior to surgery (vs mild)				
Null	0.860 (0.617–1.200)	.376		
Moderate	0.693 (0.509–0.944)	.020^∗^		
Severe	0.784 (0.599–1.026)	.076		
Intensity of pain at time of ovulation prior to surgery (vs mild)				
Null	1.126 (0.881–1.440)	.342		
Moderate	0.796 (0.534–1.186)	.262		
Severe	0.637 (0.373–1.088)	.098		
Intensity of GI symptoms prior to surgery (vs mild)				
Null	0.942 (0.698–1.271)	.695		
Moderate	0.642 (0.412–0.999)	.049^∗^		
Severe	0.774 (0.501–1.196)	.248		
Infertility				
Yes vs. no	1.377 (1.042–1.821)	.025^∗^	1.401 (1.046–1.844)	.019^∗^

CI = confidence interval, DIE = deep infiltrating endometriosis, GI = gastrointestinal, HR = hazard ratio.

∗ *P* < .05.

### 3.6 Safety outcomes

Overall, 135 (35.2%) adverse events were reported (Table 5). Menopausal symptoms, including hot flushes and sweat/night sweat, were the most common related adverse events, with an incidence of 18.0% (n = 69), followed by muscle/bone pain, agrypnia, and dizziness with incidences of 5.5% (n = 21), 2.9% (n = 11), and 1.3% (n = 5), respectively. No deaths or withdrawal cases due to adverse events occurred.

**Table 5:** Adverse events in the study population.

Adverse events	No. of AEs (person-time)	% incidence (N = 384)
All adverse events	135	35.2
Menopause symptoms (hot flash/flush, sweat/night sweat)	69	18.0
Osteoporosis (muscle pain/bone pain)	21	5.5
Agrypnia	11	2.9
Dizziness	5	1.3
Tooth ache	3	0.8
Breast lump and pain	2	0.5
Spots	2	0.5
Hand numbness	2	0.5
Edema in limbs/facial swelling	2	0.5
Chest tightness	2	0.5
Others^∗^	16	4.2

AE = adverse event.

∗ Others included headache, premature ovarian failure, pelvic infection, left lower abdomen pain, neurasthenia breakdown, hair loss, hypertension, hearing loss, decreased response, irritable, fatigue, stiff neck, atony, abnormal liver function, increased levels of thirst, and leg muscle tremors.

## 4 Discussion

In this multicenter, prospective, non-interventional study in China, treatment with triptorelin 3.75 mg once every 28 days for up to 24 weeks after conservative surgery in patients with DIE improved symptoms from baseline over the 24-month follow-up period. The improvements in pelvic pain, dysmenorrhea, pain at the time of ovulation, dyspareunia, menorrhagia, metrorrhagia, gastrointestinal symptoms, and urinary symptoms with triptorelin treatment in this study are consistent with findings from previous studies on the improvement in pain symptoms with triptorelin,^[15,16,20]^ and provide further support for the use of triptorelin after surgery for DIE. In addition, the proportions of patients with these symptoms decreased over time, particularly those with moderate and severe symptoms. The cumulative rates of improvement in individual symptoms over the 24-month period ranged from 55.1% to 83.6%.

In the present study, recurrence was evaluated based on symptoms of endometriosis. The overall 12-month cumulative rate of symptom recurrence was 12.8%, which was higher than the reported rate of 5% in a randomized study.^[21]^ However, the 24-month cumulative rate of symptom recurrence of 22.2% is similar to the endometrioma relapse rate of 21% reported in a randomized controlled trial with 5-year follow up.^[22]^ The 12-month and 24-month recurrence rates of individual symptoms were <5%, with the exception of those for dysmenorrhea (10.0% and 16.9%, respectively), which were similar to the previously reported 6-month dysmenorrhea recurrence rate of 13.3% following completion of 24 weeks of triptorelin treatment.^[23]^ Differences in study design and patient inclusion and exclusion criteria are likely to contribute to differences in symptom recurrence rates. The symptom recurrence rate gradually increased across follow up period, highlighting the importance of long-term healthcare in symptom management.

At present, there is no consensus regarding the treatment regimen for DIE, although a stepwise approach is usually suggested.^[14]^ Surgery is a common treatment option for patients with DIE.^[24]^ A previous report suggested that conservative surgery should be offered if the total excision of DIE foci is possible.^[10]^ Regarding the alleviation of pain, the benefits of surgery should always be balanced with the risk of intraoperative complications.^[25,26]^ For previously infertile women with DIE, whether to opt for in vitro fertilization treatment or surgery first can be a dilemma. Pregnancy complications, such as placenta previa, gestational hypertonia, and intrauterine growth restriction have been shown to be more common in women with DIE even after complete excision compared with women without endometriosis.^[27,28]^

Identifying risk factors for recurrence may assist early intervention and prevention. In addition to the multiple DIE locations and more severe pain symptoms identified as risk factors for recurrence in the present study, a positive family history has been shown to be a risk factor for endometrioma recurrence.^[13]^ Particular care and attention are required when managing these high-risk patients.

The overall 12-month and 24-month pregnancy rates in this study were 10.9% and 17.2%, respectively. For the subpopulation of previously infertile subjects, pregnancy rates were higher, with 18.6% at 12 months and 27.1% at 24 months. This 12-month pregnancy rate was slightly lower than that reported of 27.5% for previously infertile women with endometriosis treated with triptorelin in a randomized controlled trial (27.5%).^[21]^ However, as the study did not directly capture how many women tried to become pregnant during the 24 months follow-up, the interpretation of this observation is unclear. Furthermore, the present 24-month pregnancy rate for previously infertile patients was substantially lower than the 24-month pregnancy rate of 80% observed in previously infertile patients treated with triptorelin in the randomized controlled trial by Xue et al.^[19]^ A possible reason for this difference is the older age of patients in this study compared with the study by Xue et al (mean age, 33.4 years vs 26.7 years), as the problem of infertility is greater and the planning of pregnancy less common in older patients.

A recent randomized trial by Angioni et al^[18]^ demonstrated improvements in pelvic pain of patients with DIE with GnRH-a treatment, consistent with the present results, and also showed that the effect of GnRH-a was closely related to the treatment duration. In this study, the triptorelin treatment duration showed great variation across the study population. Most women received either 3 (30.0%) or 6 injections (39.6%). The Cox-regression model suggested that patients who were older at the time of surgery, fertile, or with history of hormonal treatment for endometriosis tended to be treated with triptorelin for longer. Thus, treatment duration is associated with patient health status, disease severity, and history of therapy.

The most common adverse effects in patients treated with triptorelin were menopause symptoms (hot flash/flush, sweat/night sweat) in agreement with results from previous studies.^[15,16,19,29]^

The key strength is that it is the first real-world study focusing on the efficacy and safety of triptorelin treatment after surgery in patients with DIE in multiple centers in China. It reflects real-world clinical practice in China and may provide clinical guidance in the future. Additionally, risk factors of symptoms recurrence might help clinical decisions for prevention of recurrence.

There are, however, several limitations. As a non-interventional study conducted in routine clinical practice, missing data are inevitable. Also, in the absence of a control group, the results of efficacy and safety can only be compared with published studies. In addition, the treatment regimen and duration were adjustable, according to routine clinical practice, which may have influenced the outcomes. Furthermore, due to the first follow-up visit was 3 months after surgery (owing to practical clinical considerations), it was difficult to determine the immediate effectiveness of surgery. Importantly, DIE has a complex phenotype and, as such, evaluation of symptom improvement alone may not capture the effects of treatment on all aspects of the disease.

It can be concluded that triptorelin following conservative surgery for DIE had a beneficial impact on pelvic pain, dysmenorrhea, ovulation pain, and dyspareunia. Over 24 months, the recurrence rate of symptoms was low and triptorelin was generally well tolerated.

## Acknowledgments

The authors thank all patients involved in the study, as well as their caregivers, care team, investigators, and research staff in participating institutions. The authors also thank Beijing Preintell Biomed Co., Ltd., who provided medical writing support, which was sponsored by Ipsen, in accordance with Good Publication Practice guidelines. Furthermore, the authors gratefully acknowledge all the investigators of research institutions for their contribution to the study: Li Hong (Remin Hospital of Wuhan University), Juxin Zhang (Henan Provincial People's Hospital), Min Hao (The Second Hospital of Shanxi University), Jianliu Wang (People's Hospital of Peking University), Ouping Huang (Jiangxi Maternal and Child Health Hospital), Hongbo Wang (Wuhan Union Hospital of Tongji Medical College of Huazhong University of Science and Technology), Jun Zhang (Beijing Anzhen Hospital of Capital Medical University), Dong Zhao (The First Maternal and Infant Hospital of Shanghai), Weidong Zhao (Anhui Provincial Cancer Hospital), Zhiqing Liang (Southwest Hospital of the Third Military Medical University), Lifang Sun (The Fourth Medical College of Peking University and Jishuitan Orthopaedic College of Tsinghua University).

## Author contributions

Libo Zhu performed the study and was a major contributor in writing the manuscript. Zheng Guan, Yan Huang, Keqin Hua, Liguo Ma, Jian Zhang, and Dazhen Yang performed the study and made a contribution to the study design and manuscript draft. Valerie Perrot and Hongbo Li analyzed and interpreted clinical data, provided feedback on the manuscript. Xinmei Zhang designed and performed the study, was a major contributor in writing the manuscript and gave final approval of the version to be submitted. All authors read and approved the final manuscript.

**Conceptualization:** Xinmei Zhang.

**Data curation:** Zheng Guan, Yan Huang, Keqin Hua, Liguo Ma, Jian Zhang, Dazhen Yang, Xinmei Zhang.

**Formal analysis:** Zheng Guan, Yan Huang, Keqin Hua, Liguo Ma, Jian Zhang, Dazhen Yang, Valerie Perrot, Hongbo Li.

**Funding acquisition:** Xinmei Zhang.

**Investigation:** Xinmei Zhang.

**Methodology:** Libo Zhu, Zheng Guan, Yan Huang, Keqin Hua, Liguo Ma, Jian Zhang, Dazhen Yang, Xinmei Zhang.

**Project administration:** Libo Zhu, Zheng Guan, Dazhen Yang.

**Resources:** Libo Zhu, Xinmei Zhang.

**Software:** Valerie Perrot, Hongbo Li.

**Supervision:** Dazhen Yang, Hongbo Li, Xinmei Zhang.

**Validation:** Valerie Perrot, Xinmei Zhang.

**Writing – original draft:** Libo Zhu.

**Writing – review & editing:** Zheng Guan, Yan Huang, Keqin Hua, Liguo Ma, Jian Zhang, Dazhen Yang, Xinmei Zhang.
